# Noisy Dueling Double Deep Q-Network algorithm for autonomous underwater vehicle path planning

**DOI:** 10.3389/fnbot.2024.1466571

**Published:** 2024-10-14

**Authors:** Xu Liao, Le Li, Chuangxia Huang, Xian Zhao, Shumin Tan

**Affiliations:** ^1^School of Mathematics and Statistics, Changsha University of Science and Technology, Changsha, Hunan, China; ^2^School of Meteorology and Oceanography, National University of Defense Technology, Changsha, Hunan, China; ^3^College of Science, Hunan University of Science and Engineering, Yongzhou, Hunan, China

**Keywords:** AUV, path planning, deep reinforcement learning, ND3QN, noisy network

## Abstract

How to improve the success rate of autonomous underwater vehicle (AUV) path planning and reduce travel time as much as possible is a very challenging and crucial problem in the practical applications of AUV in the complex ocean current environment. Traditional reinforcement learning algorithms lack exploration of the environment, and the strategies learned by the agent may not generalize well to other different environments. To address these challenges, we propose a novel AUV path planning algorithm named the Noisy Dueling Double Deep Q-Network (ND3QN) algorithm by modifying the reward function and introducing a noisy network, which generalizes the traditional D3QN algorithm. Compared with the classical algorithm [e.g., Rapidly-exploring Random Trees Star (RRT*), DQN, and D3QN], with simulation experiments conducted in realistic terrain and ocean currents, the proposed ND3QN algorithm demonstrates the outstanding characteristics of a higher success rate of AUV path planning, shorter travel time, and smoother paths.

## 1 Introduction

With the rapid advancement of artificial intelligence, the autonomous underwater vehicle (AUV) is utilized across a multitude of fields, such as environmental monitoring (Bayat et al., 2016), deep-ocean exploration (Zhang et al., 2022), seabed mapping (Ambastha et al., 2014), etc. To guarantee that an AUV can execute its missions efficiently in complex marine environments, successful path planning is the primary process. The optimization objectives of path planning encompass enhancing the success rate of AUV path planning and reducing travel time while considering energy consumption (Sun et al., 2022). However, in real ocean environments, ocean currents tend to be complex and variable, and comprehensive information about all obstacles is often unavailable. AUV can only rely on locally detectable information for path planning. Therefore, how to improve the success rate of AUV path planning and reduce the travel time as much as possible is a very challenging and crucial problem.

Over the past few decades, researchers have developed numerous path-planning algorithms, which can generally be categorized into traditional and intelligent methods (Kot, 2022). Traditional algorithms, such as Dijkstra's (Wenzheng et al., 2019) and A* (Qian et al., 2024), are well-known for their ability to find reasonably short paths in fully known environments. However, their effectiveness diminishes in unknown or dynamic environments where complete environmental information is unavailable. To address this, local path planners like the Artificial Potential Field (APF) method (Liu et al., 2020) have been employed to avoid unknown obstacles by simulating natural forces. Despite its effectiveness in certain scenarios, APF is prone to getting stuck in local minima. Alternatively, the Rapidly-exploring Random Tree (RRT) algorithm (Zeng et al., 2023) can generate collision-free paths in unknown environments through random sampling, but this randomness often leads to non-smooth paths, increasing the control difficulty and energy consumption of AUVs. Karaman and Frazzoli (2011) propose the rapid-exploring random tree star (RRT*) algorithm, which modifies the node expansion strategy. It effectively solves the suboptimal trajectory problem of RRT, planning smoother paths and increasing the success rate of path planning. Fu et al. (2019) apply the RRT* algorithm to AUV path planning and approach an optimal path with less travel time more quickly in varying terrain and scattered floating obstacles. The BI-RRT* algorithm proposed by Fan et al. (2024) exhibits superior path planning capabilities to the RRT* algorithms by extending the obstacle region, employing a bidirectional search strategy. Nevertheless, when obstacles and other factors in the environment change, the RRT* algorithm needs to re-plan the path and is not sufficiently adaptable to different environments (Khattab et al., 2023).

Reinforcement learning (RL) is an emerging intelligent method that offers a more flexible and adaptive solution for AUV path planning. It mimics the human learning process by letting agents continuously engage with the environment, gain experience, and discover the optimal strategy. The learning process is guided by reward functions, making this approach particularly suitable for executing specific tasks [e.g., selecting actions that follow ocean currents (Li et al., 2023)]. After trained, RL can apply their learned knowledge to different unknown environments. Deep Q-Network (DQN) (Mnih et al., 2015) is a classical reinforcement learning algorithm that combines Q-learning (Soni et al., 2022) and deep neural networks (Krizhevsky et al., 2012) to address problems with continuous state spaces. Yang et al. (2023b) successfully employed DQN to achieve efficient path planning with varying numbers of obstacles, improving the success rate of AUV path planning across different environments. Zhang and Shi (2023) combine DQN with Quantum Particle Swarm Optimization to create the DQN-QPSO algorithm. By considering both path length and ocean currents in the fitness function, this algorithm effectively identifies energy-efficient paths in underwater environments. Despite significant advancements, DQN tends to overestimate Q-values during training. Hasselt et al. (2016) propose the Double DQN (Double Deep Q-Network) to address the issue in DQN and enhance the algorithm's performance. Chu et al. (2023) improve the DDQN algorithm and used the NURBS algorithm to smooth the path. Yang et al. (2023b) propose an N-step Priority Double DQN (NPDDQN) path planning algorithm, to make better use of high-value experience to speed up the convergence of the training process. Wang et al. (2016) propose the Dueling Double Deep Q-Network (D3QN), which combines Double DQN and Dueling DQN. Xi et al. (2022) optimize the reward function within the D3QN algorithm to account for ocean currents. Although this adaptation enables the planning of paths with shorter travel times, the resulting paths are not always smooth. While these RL algorithms have made progress in AUV path planning, they still have limitations in exploration. The uncertain environment requires the agent to not only utilize existing knowledge but also to continuously explore unknown areas to avoid getting stuck in a suboptimal path. DQN and D3QN algorithms typically use an ε-greedy strategy for action selection, where actions are chosen randomly with a probability of ε, and 1-ε to choose optimal action by the current model. The approach might lead to inadequate exploration in the initial phases of learning and an excessive degree of exploration as learning progresses (Sharma et al., 2017).

To address the exploration deficiencies caused by the ε-greedy strategy, some researchers have proposed the ε-decay strategy (Astudillo et al., 2020). This method initializes with a high ε at the outset of the reinforcement learning training, prompting the agent to engage in random actions and thoroughly investigate the environment. As training progresses, the ε value gradually decreases, allowing the agent to rely more on learned experiences for decision-making. Although this approach is successful, it necessitates manual adjustment of parameters and might not guarantee enough exploration in the final stages of training. In 2015, Fortunato et al. (2017) introduce the Noisy DQN algorithm. They introduced learnable noise into the DQN neural network parameters, creating what is known as a noisy network. It allows the agent to maintain a certain level of exploration throughout the training process, which enhances the algorithm's adaptability to environmental changes and helps in obtaining better policies. The Noisy DQN method has succeeded significantly in various reinforcement learning applications (Gao Q. et al., 2021; Cao et al., 2020; Harrold et al., 2022). Inspired by the above discussion, we introduce the noisy network into the D3QN algorithm and combine it with an ε-decay strategy. Additionally, we modify the reward function to comprehensively account for various requirements, proposing a novel AUV path planning algorithm named the Noisy Dueling Double Deep Q-Network (ND3QN) algorithm. The main contributions of this study are as follows:

(1) By incorporating noisy networks, the ND3QN algorithm can dynamically adjust the level of exploration, preventing premature convergence to local optima and improving the algorithm's robustness and its ability to generalize. Meanwhile, the ND3QN algorithm considers factors such as distance, obstacles, ocean currents, path smoothness, and step count, facilitating the AUV to find smoother and less time-consuming paths.

(2) We establish a range sonar model to obtain information about local obstacles and utilize real ocean current and terrain data from the southern Brazilian, providing a more realistic simulation of the marine environment.

(3) The ND3QN algorithm significantly enhances the path-planning performance of AUV in complex environments, achieving about 93% success rate in path planning, which is a 4%–11% improvement over the RRT*, DQN, and D3QN algorithms, with a 7%–11% reduction in travel time and 55%–88% improvement in path smoothness.

The remainder of this paper is outlined as follows: Section 2 briefly introduces the AUV motion model, sonar model, basics of reinforcement learning, and the D3QN algorithm. Section 3 explains the ND3QN algorithm in detail. Section 4 validates the effectiveness and generality of the ND3QN algorithm through simulation experiments, comparing it with traditional RRT*, DQN, and D3QN algorithms. Section 5 concludes this work.

## 2 Preliminaries

In this part, we initially develop the motion and sonar models of the AUV, followed by an introduction to reinforcement learning, and conclude with the presentation of the basic framework of the D3QN algorithm.

### 2.1 AUV motion model

**Figure 1:**
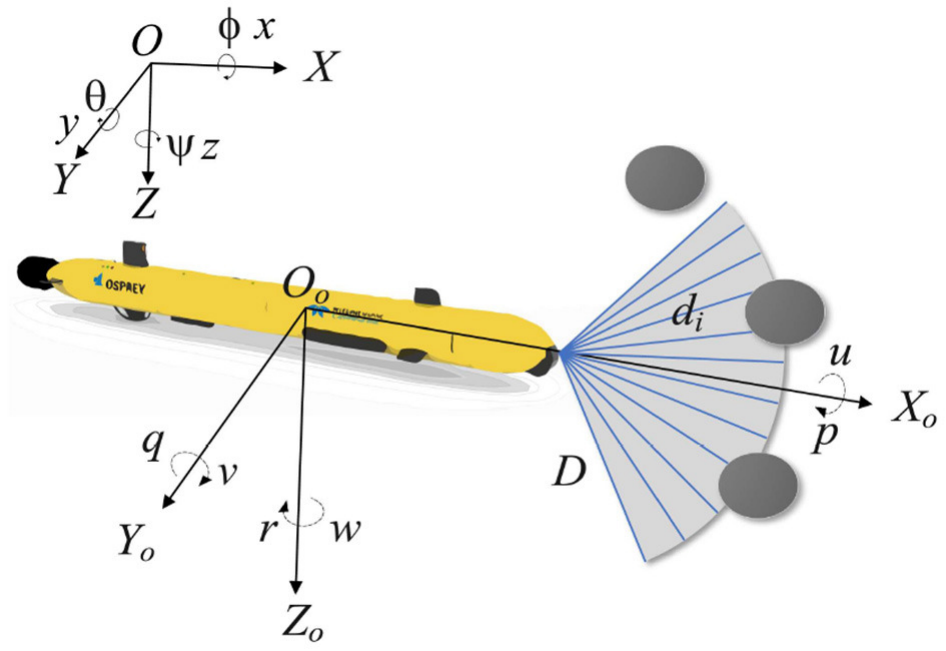
Schematic representation of the variables of the AUV motion model and the sonar model.

The position vector Θ = [*x, y, z*] and orientation vector Ψ = [ϕ, θ, ψ] of the Autonomous Underwater Vehicle (AUV) can be ascertained within the Earth's fixed coordinate system. Here, *x*, *y*, and *z* denote the spatial coordinates of the AUV, while ϕ, θ, and ψ denote the roll, pitch, and yaw angles, respectively. In the body-based coordinate system, the velocity vector of the AUV's motion in each dimension is expressed as **v** = [*u, v, w, p, q, r*], where *u*, *v*, and *w* represent the surge, sway, and heave velocities, and *p*, *q*, and *r* denote the rates of roll, pitch, and yaw, respectively (Okereke et al., 2023). The specifics of these variables are delineated in Figure 1. Assuming that the gravitational force and buoyancy are equal, the conventional kinematic equations for an AUV can be streamlined as follows (Li J. et al., 2021):


(1) $$\begin{array}{l} \{\begin{array}{l} ẋ=u\cos\psi \cos\theta +v\left(\cos\psi \cos\theta \sin\varphi -\sin\psi \cos\varphi \right)\\ +w\left(\cos\psi \cos\theta \cos\varphi +\sin\psi \sin\varphi \right)\\ ẏ=u\sin\psi \cos\theta +v\left(\sin\psi \sin\theta \sin\varphi +\cos\psi \cos\varphi \right)\\ +w\left(\sin\psi \sin\theta \cos\varphi -\cos\psi \sin\varphi \right)\\ ż=-u\sin\theta +v\cos\theta \sin\varphi +w\cos\theta \cos\varphi \\ \dot{\varphi }=p+q\sin\varphi \tan\theta +r\cos\varphi \tan\theta \\ \dot{\theta }=q\cos\varphi -r\sin\varphi \\ \dot{\psi }=q\sin\varphi /\cos\theta +r\cos\varphi /\cos\theta . \end{array} \end{array}$$

In a 2D environment, the effects of the AUV's roll and pitch can be neglected (Song et al., 2016), so ϕ, θ and *w* are zero, the simplified two-dimensional kinematic model is represented as follows:


(2) $$\begin{array}{l} \{\begin{array}{l} ẋ=u\cos\psi -v\sin\psi \\ ẏ=u\sin\psi +v\cos\psi \\ \dot{\psi }=r. \end{array} \end{array}$$

This simplified model effectively describes the AUV's planar motion, facilitating trajectory planning and control design.

### 2.2 Sonar model

During underwater missions, AUV frequently encounters complex environments such as reefs, schools of fish, and unknown obstacles. To navigate safely, AUV relies on sensors to detect their surroundings and provide data to the planning algorithm. Ranging sonar (Wang and Yang, 2013), known for its simplicity and low cost, is the most widely used underwater detection device. It emits a conical beam and calculates the distance to obstacles by measuring the time difference between transmitted and received beams. When multiple ranging sonars are combined into a multi-beam sonar, they cover a sector-shaped area in front of the AUV.

We assume that the bow of the AUV is equipped with 12 ranging sonars, each with an aperture angle of 10°, providing a horizontal coverage span from −60° to 60°. Figure 1 shows the detection area, where *D* is the maximum detection range. *d*_*i*_ is the distance to an obstacle in the beam direction. If no obstacles are detected by one of the ranging sonars, then *d*_*i*_ = *D*. The detected distances are recorded in a 1x12 matrix *M* = [*d*_1_, *d*_2_, …, *d*_12_].

### 2.3 Reinforcement learning

**Figure 2:**
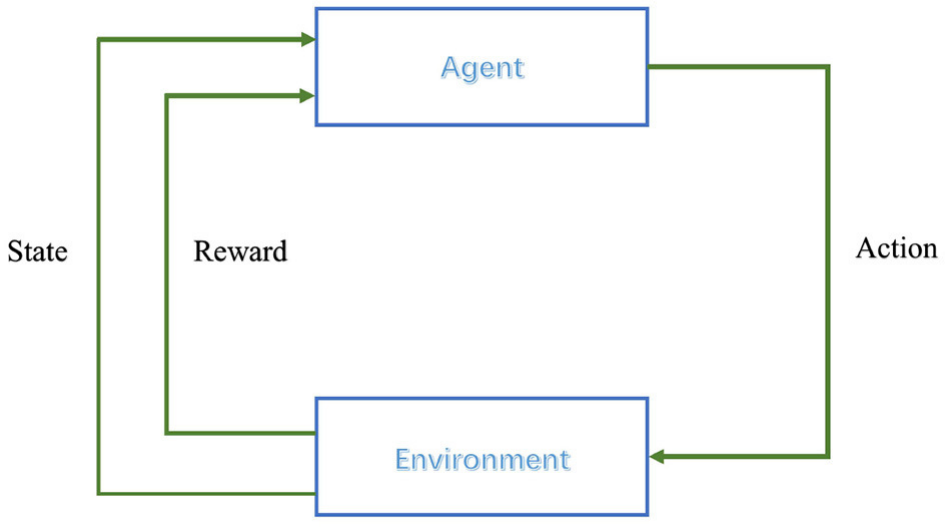
Reinforcement learning control process.

Reinforcement learning relies on the framework of Markov Decision Processes (MDPs) to model the interaction between an agent and its environment (Alvarez et al., 2004). MDPs are defined by five key components: the state space *S*, the action space *A*, the state transition probabilities *P*, the reward function *R*, and the policy π(*a*|*s*), which represents the probability of taking action *a* in state *s* (Alvarez et al., 2004). In each iteration, the agent selects an action *a*_*t*_ based on its current policy π_*t*_, which influences the state transition of the environment. This leads to a new state *s*_*t*+1_ and an associated reward *r*_*t*_ from the environment, resulting in an interaction experience (*s*_*t*_, *a*_*t*_, *r*_*t*_, *s*_*t*+1_) (Hossain et al., 2023). By gathering these experiences, the agent gradually enhances and refines its policy. The decision-making process is illustrated in Figure 2.

The cumulative discounted reward (Knox and Stone, 2012) represents the total reward that can be obtained over all future time steps after taking action in a given state. In the case of finite time steps, it can be expressed as:


(3) $$\begin{array}{l} U_{t}=r_{t}+\gamma r_{t+1}+\gamma^{2}r_{t+2}+\cdots +\gamma^{T-t}r_{T}=\overset{T-t}{\underset{k=0}{\sum }}\gamma^{k}r_{t+k}, \end{array}$$

where γ denotes the discount factor used for calculating the present value of future rewards, which takes a value between 0 and 1. *U*_*t*_ accumulates all future rewards from time step t to the terminal time step *T*.

In reinforcement learning, the action-value function and state-value function are two central concepts. The action-value function *Q*_π_(*s*_*t*_, *a*_*t*_) evaluates the value of taking an action *a* in state *s* and later acting according to strategy π. The state-value function *V*_π_(*s*_*t*_), on the other hand, does not depend on *a* specific action but rather evaluates the expected payoff that can be obtained when starting from state *s* and always following strategy π (Li W. et al., 2021). They are formulated as follows:


(4) $$\begin{array}{l} Q_{\pi }\left(s_{t},a_{t}\right)={𝔼}_{a_{t}\sim \pi \left(a_{t}|s_{t}\right)}\left[U_{t}|s_{t},a_{t}\right], \end{array}$$

(5) $$\begin{array}{l} V_{\pi }\left(s_{t}\right)={𝔼}_{a_{t}\sim \pi \left(\cdot |s_{t}\right)}\left[U_{t}|s_{t}\right]. \end{array}$$

To estimate the value function in a continuous state space, the Deep Q-Network (DQN) incorporates neural networks, which use *Q*_π_(*s*_*t*_, *a*_*t*_; δ) to approximate the estimate of *Q*_π_(*s*_*t*_, *a*_*t*_), where δ denoting the parameters of the neural network (Gao Y. et al., 2021). DQN further includes a target network $Q_{\pi }^{\prime }\left(s_{t+1},a_{t+1};\delta^{\prime }\right)$, whose parameters are periodically updated from those of the current network, to estimate the maximum value of the subsequent state-action pair. Following is the computation of the DQN loss function:


(6) $$\begin{array}{l} \text{Loss}=𝔼\left[{\left(r+\gamma \underset{a_{t+1}}{\max}Q_{\pi }^{\prime }\left(s_{t+1},a_{t+1};\delta^{\prime }\right)-Q_{\pi }\left(s,a;\delta \right)\right)}^{2}\right]. \end{array}$$

The introduction of the target network in DQN enhances its efficiency and convergence, reducing variance during training and stabilizing the learning process.

### 2.4 Dueling Double Deep Q-Network

Merging the advantages of Double DQN with those of Dueling DQN results in the formation of the Dueling Double Deep Q-Network (D3QN) algorithm. This integration addresses the overestimation bias typically found in the conventional DQN, thereby enhancing the learning process's overall performance and stability. Double DQN separates the action selection from the target Q-value computation by identifying the action with the highest Q-value in the current Q network and then using this action to calculate the target Q-value in the target Q network, effectively reducing the risk of overestimation (Hasselt et al., 2016). Here is the definition of the loss function:


(7) $$\begin{array}{l} \text{Loss}=\\ 𝔼\left[{\left(r+\gamma Q_{\pi }^{\prime }\left(s_{t+1},\arg\underset{a_{t+1}}{\max}Q_{\pi }\left(s_{t+1},a_{t+1};\delta \right);\delta^{\prime }\right)-Q_{\pi }\left(s,a;\delta \right)\right)}^{2}\right], \end{array}$$

where $\arg\underset{a_{t+1}}{\max}Q_{\pi }\left(s_{t+1},a_{t+1};\delta \right)$ denotes the selection of an action *a*_*t*+1_ from the current Q network that maximizes the Q value in state *s*_*t*+1_.

The Dueling DQN algorithm decomposes the Q-value function into two separate components: one representing the state value and the other encapsulating the advantage. The state value function estimates the expected return for a particular state, while the advantage function quantifies the benefit of taking a specific action compared to the average performance in that state. This separation enhances the accuracy of state value assessments and the evaluation of action benefits, leading to improved model efficiency and performance. The Q-value in Dueling DQN is computed as follows:


(8) $$\begin{array}{l} Q_{\pi }\left(s,a;\delta_{s},\delta_{a}\right)=\left(A\left(s,a;\delta_{a}\right)-\frac{1}{\left|A\right|}\underset{a_{i}\in A}{\sum }A\left(s,a_{i};\delta_{a}\right)\right)+V_{\pi }\left(s;\delta_{s}\right), \end{array}$$

where *V*_π_(*s*; δ_*s*_) denotes the state value function, *A*(*s, a*; δ_*a*_) represents the advantage function, and |A| denotes the total number of possible actions. By integrating the enhancements from these two algorithms, we can formulate the corresponding loss function for D3QN as follows:


(9) $$\begin{array}{l} \text{Loss}=𝔼[(r+\gamma Q_{\pi }^{\prime }(s_{t+1},\arg\underset{a_{t+1}}{\max}Q_{\pi }(s_{t+1},a_{t+1};\delta_{s},\delta_{a});\delta_{s}^{\prime },\delta_{a}^{\prime })\\ \;-Q_{\pi }(s,a;\delta_{s},\delta_{a}){)}^{2}] \end{array}$$

D3QN has demonstrated superior performance in various applications compared to traditional DQN, establishing it as a leading algorithm in reinforcement learning (Gök, 2024).

## 3 Methods

This section provides a detailed introduction to the ND3QN algorithm. Initially, we describe the environmental state variables, including AUV position and orientation information, obstacle information, and ocean current data. Subsequently, we expand on the action space and describe the state transition method. Following this, we elaborate on the composite reward function, which accounts for factors such as ocean currents, obstacles, and turning constraints. Lastly, we introduce a noise network based on the D3QN algorithm.

### 3.1 Environmental states

In the realm of reinforcement learning research and applications, environmental state variables form the foundation of an agent's perception of the surrounding environment. These variables constitute a set of observational data, thereby providing a comprehensive representation of the agent's context, which is crucial for the agent to make effective decisions (Sutton and Barto, 2018). In the underwater operational environment of an AUV, the environmental state variables should encompass the AUV's position and orientation information, as well as external elements like ocean currents and obstacles. In this study, the AUV's local environmental information is represented by the state variables $S=\left[{\vec{\xi }}_{xy},\psi ,{\vec{\nabla }}_{\text{cur}},M\right]$, where ${\vec{\xi }}_{xy}$ denotes position information, ψ represents heading angle information, ${\vec{\nabla }}_{\text{cur}}$ indicates ocean current information, and *M* denotes the detection range matrix.

#### 3.1.1 Position orientation information

To enhance the model's ability to generalize, we convert absolute position coordinates into relative position vectors. Assuming the current position coordinates are *P*(*x, y*) and the goal position is *P*_goal_(*x*_*goal*_, *y*_*goal*_), the vector representation of the current position and goal coordinates is given as:


(10) $$\begin{array}{l} {\vec{\xi }}_{xy}=\left(x_{goal}-x,y_{goal}-y\right). \end{array}$$

The AUV's decision-making will be influenced by its attitude differences, even when it is in the same circumstance. Therefore, we incorporate the AUV's heading angle ψ as attitude information into the environmental state variable.

#### 3.1.2 Information on external environment

In complex ocean environments, the movement of an AUV is influenced by ocean currents. In real data formats, ocean current information is typically provided as gridded data. Therefore, interpolation is needed to estimate the discrete ocean current data. The ocean current value $\vec{\nabla }\text{cur}$ at *P*(*x, y*) can be interpolated from the current values at its four neighboring grid points *P*_*oj*_ (*x*_*oj*_, *y*_*oj*_), where *oj* = 1, 2, ..., 4, as follows:


(11) $$\begin{array}{l} \vec{\nabla }\text{cur}=\frac{\sum \vec{\nabla }{\text{cur}}_{oj}\cdot L_{euc}\left(P,P_{oj}\right)}{\sum L_{euc}\left(P,P_{oj}\right)}, \end{array}$$

(12) $$\begin{array}{l} L_{euc}\left(P,P_{oj}\right)=\sqrt{{\left(x-x_{oj}\right)}^{2}+{\left(y-y_{oj}\right)}^{2}}, \end{array}$$

where *L*_*euc*_(*P, P*_*oj*_) defines the Euclidean distance between two points *P*(*x, y*) and *P*_*oj*_ (*x*_*oj*_, *y*_*oj*_).

The detection range matrix *M* = [*d*_1_, *d*_1_, …, *d*_12_] can be obtained through a sonar ranging model, allowing the AUV to perceive detailed information about surrounding obstacles.

### 3.2 Action space and state transition function

**Figure 3:**
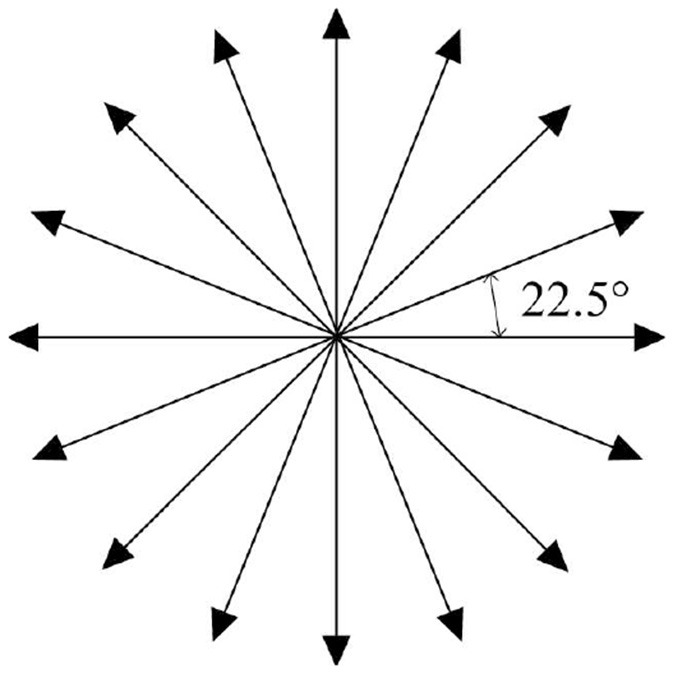
Action space.

Some of the existing AUVs can only rotate at fixed angle increments instead of arbitrary angles during navigation, as their steering rudders are limited by factors such as mechanical structure, motor characteristics, and control system. Therefore, in this paper, the navigation direction of AUV is designed as a discrete action. To broaden the range of directional options available for an AUV, we have discretized its horizontal movements into 16 distinct actions, *a* = [*a*_1_, *a*_2_, *a*_3_, ..., *a*_16_], each separated by an angle of 22.5, as illustrated in Figure 3. Compared to the existing options of 6 (Xi et al., 2022) or 8 (Yang et al., 2023a) actions, the availability of 16 actions offers a finer degree of directional precision, enabling the AUV to navigate complex underwater environments better. For instance, when encountering complex underwater obstacles or ocean currents, the AUV can select actions more congruent with its desired heading, facilitating smoother and more efficient path planning.

Based on the selected action *a* at the current position, the subsequent position *P*′(*x*′, *y*′) is determined as follows:


(13) $$\begin{array}{l} \left[\begin{array}{c} x^{\prime }\\ y^{\prime } \end{array}\right]=\left[\begin{array}{c} x\\ y \end{array}\right]+\left(V_{\text{AUV}}\left[\begin{array}{c} cos\left(a\right)\\ sin\left(a\right) \end{array}\right]+\left[\begin{array}{c} u\\ v \end{array}\right]\right)\Delta t, \end{array}$$

where *V*_AUV_ denotes the velocity of AUV, the east-west component of the ocean current ${\vec{\nabla }}_{\text{cur}}$ is shown by *u*, while the north-south component is indicated by *v*, and Δ*t* represents the control time interval.

Path planning involves generating a trajectory from a starting point to a destination, which can be represented as a sequence of waypoints. An AUV initiates its journey from the starting point and selects its current action based on discrete action decision-making. Subsequently, by executing the chosen action in conjunction with the position of the current waypoint, the AUV determines the location of the subsequent waypoint. This iterative process continues until the AUV reaches the final destination, thereby compiling a series of waypoints that collectively constitute a complete path.

### 3.3 Reward function

Evaluating an agent's actions is paramount, with the reward function being one of the key determinants in the success and efficiency of reinforcement learning algorithms. Especially in environments with highly complex states and actions, reliance on a single reward often hinders the agent from effectively learning the optimal policy (Tang et al., 2024). Consequently, after a comprehensive consideration of the motion control characteristics, environmental obstacles, and ocean current influences during AUV navigation, we have designed a composite reward function. This function encompasses distance reward, obstacle avoidance reward, ocean current reward, path smoothness reward, and step reward.

#### 3.3.1 Distance reward

The distance reward guides the AUV to approach the goal point. A fixed reward *R*_goal_ is awarded when the AUV reaches the goal point, and *T*_*done*_ = 1. In contrast, we assess the action in terms of the difference between the distance from the goal point at this time and the distance from the goal point at the previous instant if the AUV does not reach the goal point, and (*T*_*done*_ = 0). The following is the formula used to calculate the distance reward:


(14) $$\begin{array}{l} R_{\text{dis}}=L_{euc}\left(P_{t},P_{\text{goal}}\right)-L_{euc}\left(P_{t+1},P_{\text{goal}}\right), \end{array}$$

where the distance at time *t* between the current position and the goal is represented by *L*_*euc*_(*P*_*t*_, *P*_goal_). If *R*_dis_ is positive, it indicates that the AUV is progressing toward the goal direction.

#### 3.3.2 Obstacle avoidance reward

In the detection distance matrix derived from the sonar model, obstacles detected across various directions exert varying degrees of influence on AUV. Those obstacles directly ahead, within the bow direction, exert the most significant impact on the AUV's navigation, with this impact diminishing progressively as the angle measured from the bow direction increases. The influence of detected obstacles in different directions is quantified by the parameter ω(*i*), which is determined through the following computation:


(15) $$\begin{array}{l} \omega \left(i\right)=e^{-|i-\left(n-1\right)/2|},\;i=1,2,3,\ldots ,n, \end{array}$$

where *i* denotes the identifier of the sonar sensor, with the current heading index being (*n* − 1)/2, and *n* indicates the count of detection sonars. The AUV's detection weight is maximum in the current heading direction and decreases gradually toward both sides. Combining the obstacle information and the degree of impact in the detection matrix *M* = [*d*_1_, *d*_1_, …, *d*_12_], the obstacle penalty information is defined as:


(16) $$\begin{array}{l} R_{\text{obs}}=\overset{n}{\underset{i=1}{\sum }}\left(1-\frac{d_{i}}{D}\right)\cdot \omega \left(i\right), \end{array}$$

where *d*_*i*_ represents the separation between the AUV detected by the *i*-th sonar sensor and the obstacle, *d*_*i*_ ∈ *M*, with *D* denoting the detection range.

When no obstacles are detected within the scanning range, no penalty is incurred. Conversely, when an obstacle is detected within the range, a penalty *R*_obs_ is applied. A smaller value of *R*_obs_ indicates that the chosen direction of movement is more likely to result in a collision with an obstacle. Furthermore, in the event of a direct collision with an obstacle, a fixed penalty *R*_col_ is imposed.

#### 3.3.3 Ocean current reward

In the course of AUV navigation, ocean currents are an indispensable external factor that directly impacts the AUV's speed and energy consumption. To effectively utilize ocean currents for reducing both travel time and energy consumption, we introduce a reward function related to ocean currents:


(17) $$\begin{array}{l} R_{\text{cur}}=\cos\left(\theta_{c}\right)\frac{V_{\text{cur}}}{V_{\text{AUV}}}, \end{array}$$

where θ_*c*_ indicates the orientation difference between the ocean current's flow and the AUV's direction of movement. A larger value of *R*_cur_ indicates a closer alignment between the AUV's motion direction and the ocean current direction, as well as a higher degree of utilization of the ocean current's strength.

#### 3.3.4 Path smoothness reward

Due to the complexity of AUV control underwater, frequent turning can lead to deviations from the planned trajectory. To maintain trajectory stability, we aim to minimize the change between consecutive actions. We achieve this by introducing a reward function *R*_sta_, defined as:


(18) $$\begin{array}{l} R_{\text{sta}}=\cos\left(\left|a_{t-1}-a_{t}\right|\right), \end{array}$$

where *a*_*t*−1_ signifies the action taken in the preceding time step, a higher value of *R*_sta_ suggests a smaller change in action, thereby conferring a greater positive reward. This reward incentivizes the AUV to execute smoother actions, thereby mitigating the occurrence of abrupt turns and sharp maneuvers.

#### 3.3.5 Step reward

To avoid unnecessary back-and-forth movements of the AUV underwater and to encourage the selection of relatively shorter paths, we introduce a fixed negative penalty *R*_step_ at each step.

#### 3.3.6 Comprehensive reward

The comprehensive reward function is formulated as the aggregated, weighted combination of all the aforementioned reward functions:


(19) $$\begin{array}{l} R_{\text{total}}=k_{1}R_{\text{dis}}+k_{2}R_{\text{obs}}+k_{3}R_{\text{cur}}+k_{4}R_{\text{sta}}+k_{5}R_{\text{step}}+T_{\text{done}}R_{\text{goal}}, \end{array}$$

where *k*_*i*_ are the weighting factors, and *T*_*done*_ = 0 or 1, indicates the status of reaching the goal point.

The total reward function, compared to a single reward function, can better accommodate complex task requirements. In particular, due to the obstacle avoidance reward component, the closer the obstacle is to the forward direction of the AUV, the greater the negative penalty imposed. This design helps the AUV detect and avoid obstacles in advance, accelerating the learning process. Additionally, the path smoothness reward encourages the AUV to select actions with smaller turning angles, thereby preventing frequent turns and sharp maneuvers. This approach enhances the AUV's navigational stability and smoother trajectory. To sum up, the composite reward function designed in this study comprehensively accounts for the operational efficiency and safety of the AUV, leveraging information on ocean currents and obstacles to guide the agent toward more time-efficient and smooth paths.

### 3.4 Path planning algorithm

In conventional deep reinforcement learning, the ε-greedy strategy (Dann et al., 2022) is commonly employed to explore the environment. This strategy involves taking actions at random with a constant probability ε to facilitate exploration and selecting the action currently deemed optimal with a probability of 1 − ε for exploitation purposes. Nonetheless, an over-reliance on experience may result in convergence to local optima, precluding the identification of a global optimum. Conversely, excessive exploration can prolong training duration and diminish efficacy. To tackle this challenge, we propose a novel strategy that balances exploration and exploitation by decaying the probability of selecting random actions and incorporating noisy networks.

We incorporate noisy networks into the D3QN algorithm framework to augment the algorithm's exploration capabilities. Noisy networks introduce parameterized noise into the neural network, which facilitates a more comprehensive exploration of the state space. By introducing noisy parameters, the network can experiment with different combinations of weights and biases during each update, thus enhancing its exploration of the environment and uncovering new, previously unknown state-action pairs. Typically, a fully connected layer receives *p* inputs and produces *q* outputs, which can be represented as *Y* = *WX* + *B*. Here, *W* denotes the matrix of weights, *X* is the input vector to the layer, and *B* is the bias vector. We substitute the parameters *w* and *b* in the neural network with μ + σ ∘ ξ, where the notation ∘ denotes element-wise multiplication (Yang et al., 2021). The equivalent noisy linear layer is formulated as follows:


(20) $$\begin{array}{l} Y=\left(\mu^{W}+\sigma^{W}\circ \xi^{W}\right)X+\mu^{B}+\sigma^{B}\circ \xi^{B}, \end{array}$$

where μ^*B*^ + σ^*B*^ ∘ ξ^*B*^ and μ^*W*^ + σ^*W*^ ∘ ξ^*W*^ supplant *B* and *W*, respectively. The elements μ^*B*^, μ^*W*^, and σ^*W*^, σ^*B*^ denote the learned mean and standard deviation values through empirical data. The variables within ξ^*W*^ and ξ^*B*^ are independently sampled from a standard normal distribution, *N*(0, 1). And then the action value function is denoted as *Q*(*s, a*, ξ; μ, σ).

**Figure 4:**
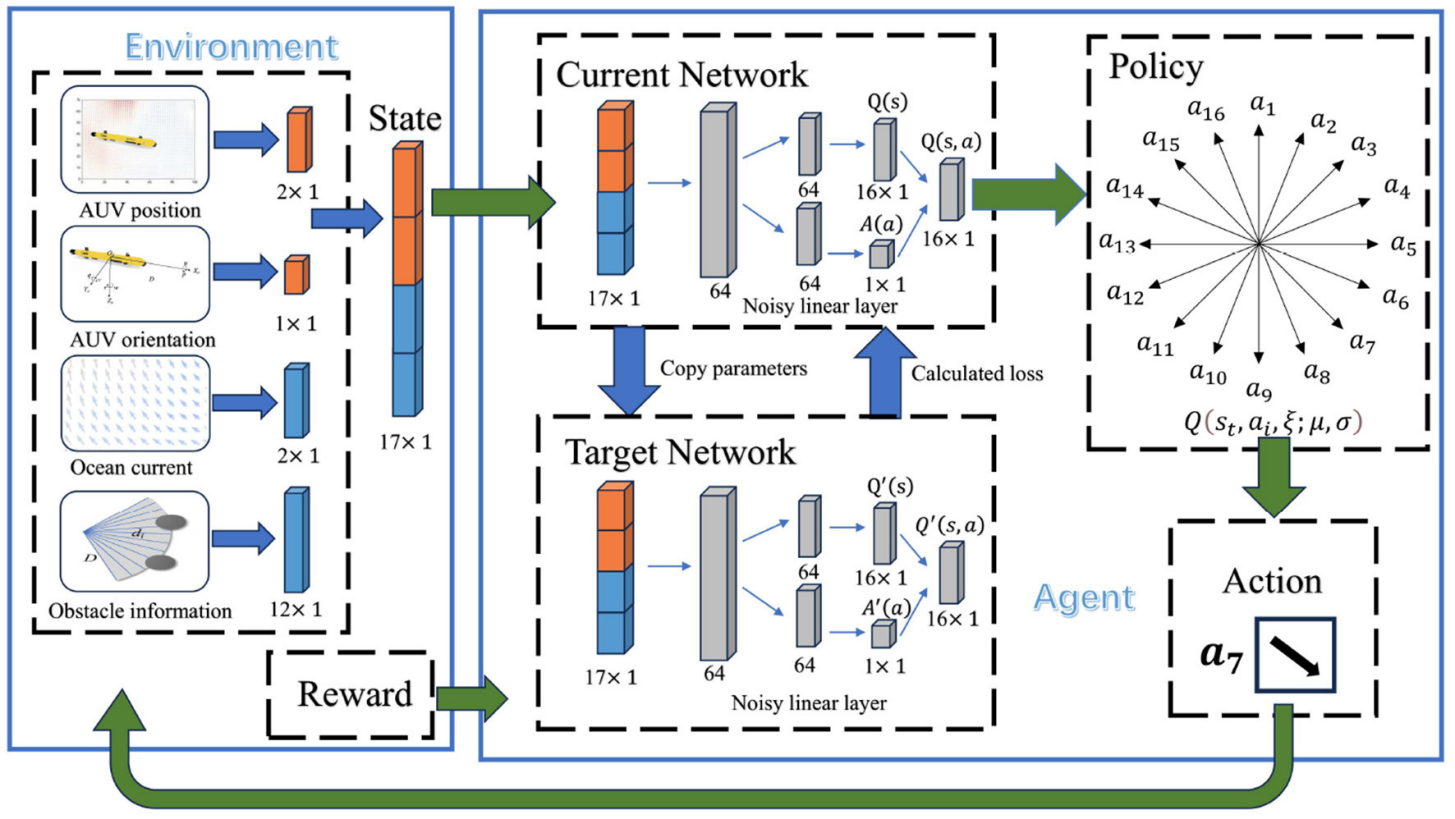
Decision-making process of the ND3QN algorithm.

Noisy networks are a pivotal element within the ND3QN algorithm, contributing to the following dimensions: (1) Increased exploration: By incorporating trainable noise into the neural network parameters, noisy networks ensure that AUV retains a consistent level of exploratory capacity during the training phase. This facilitates the AUV's exploration of diverse strategies and the discovery of novel state-action pairs, preventing convergence to suboptimal solutions. (2) Accelerating learning pace: The noisy networks enhance the exploratory randomness during the early stages of training, allowing AUV to rapidly identify strategies associated with higher rewards, thereby expediting the learning process. (3) Enhancing algorithm robustness and generalizability: The noisy networks allow the AUV to keep exploring in a variety of environments, which makes the algorithm more reliable and usable in more situations. This versatility enables the ND3QN algorithm to more effectively adapt to differing marine scenarios and to identify superior navigation paths. Eventually, a flowchart of the AUV's decision-making for each step of the action can be obtained, as shown in Figure 4.

Simultaneously, we also integrated the ε-decay strategy into the training process. Decaying the exploration probability of random actions means starting with a high exploration rate and gradually decreasing it until it converges to a lower value. This process is achieved by setting an initial exploration rate, a final exploration rate, and a decay function. The specific formula is as follows:


(21) $$\begin{array}{l} \varepsilon_{t}=\varepsilon_{\text{final}}+\left(\varepsilon_{\text{initial}}-\varepsilon_{\text{final}}\right)\times e^{-\frac{C_{step}}{\Gamma }}, \end{array}$$

where ε_initial_ represents the initial degree of exploration, ε_final_ denotes the final level of exploration, *C*_step_ is the global step count, and Γ represents a constant parameter that governs the pace at which the exploration rate evolves throughout training iterations. The action selection is then as follows:


(22) $$\begin{array}{l} a=\{\begin{array}{ll} \text{randomly chosen }a\in A, & \text{with probability }\varepsilon_{t},\\ \underset{a}{\text{argmax }}Q_{\pi }\left(s_{t},a,\xi ;\mu ,\sigma \right), & \text{with probability}1-\varepsilon_{t}. \end{array} \end{array}$$

The loss of the ND3QN formed by combining the D3QN and the noisy network is calculated as follows:


(23) $$\begin{array}{l} \text{Loss}\\ =𝔼[(r+\gamma *Q_{\pi }^{\prime }(s_{t+1},\arg\underset{a_{t+1}}{\max}Q_{\pi }(s_{t+1},a_{t+1},\xi ;\mu ,\sigma ),\xi^{\prime };\mu^{\prime },\sigma^{\prime })\\ \;-Q_{\pi }(s_{t},a,\xi ;\mu ,\sigma ){)}^{2}] \end{array}$$

ND3QN introduces the noisy network approach to D3QN while using the ε-decay mechanism for action selection. This allows the network to automatically adjust the degree of exploration during training, while the ε-decay strategy provides additional randomness to avoid local optima. This combination enhances the algorithm's exploratory prowess, facilitating a more effective equilibrium between exploration and exploitation throughout training, thereby enhancing the algorithm's efficacy and robustness. The step-by-step procedure of ND3QN is delineated in Algorithm 1.

**Algorithm 1:**
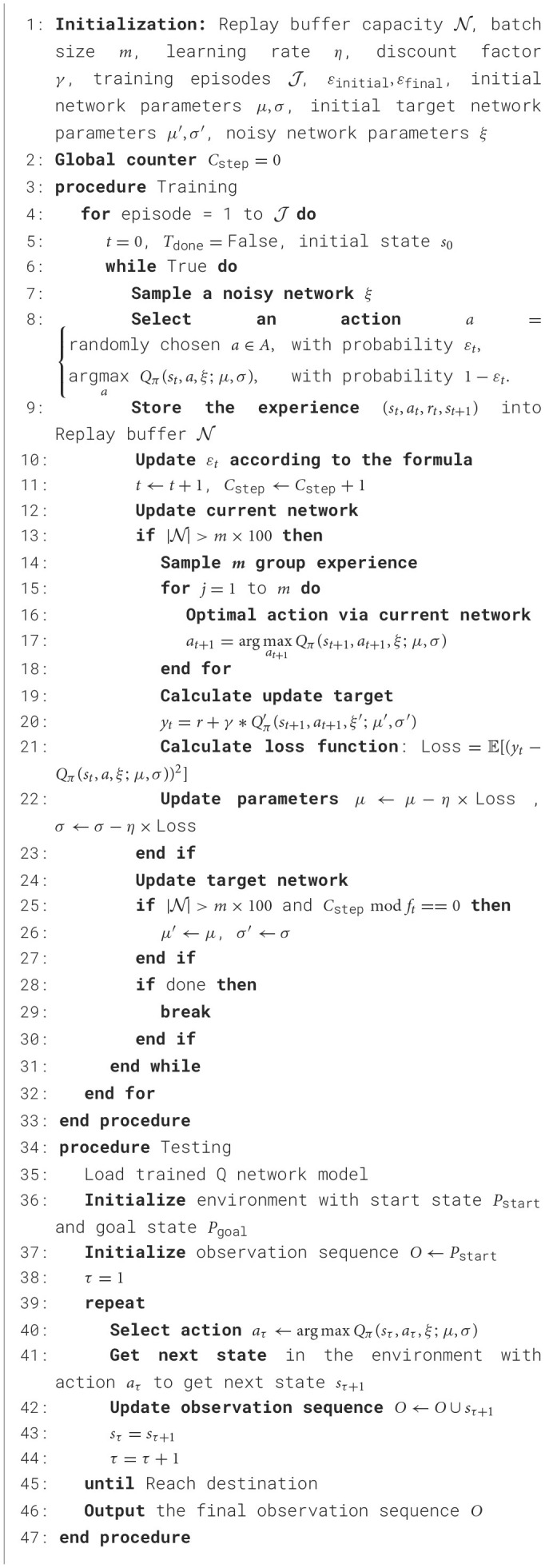
ND3QN algorithm with training and testing procedures.

## 4 Experiments

In this section, we conduct a suite of simulated experiments for AUV path planning, utilizing the proposed ND3QN algorithm with partial real-world ocean current data and topographical information. The outcomes of the experiments validate the efficacy of the proposed algorithm.

### 4.1 Environmental settings and evaluation indicators

**Figure 5:**
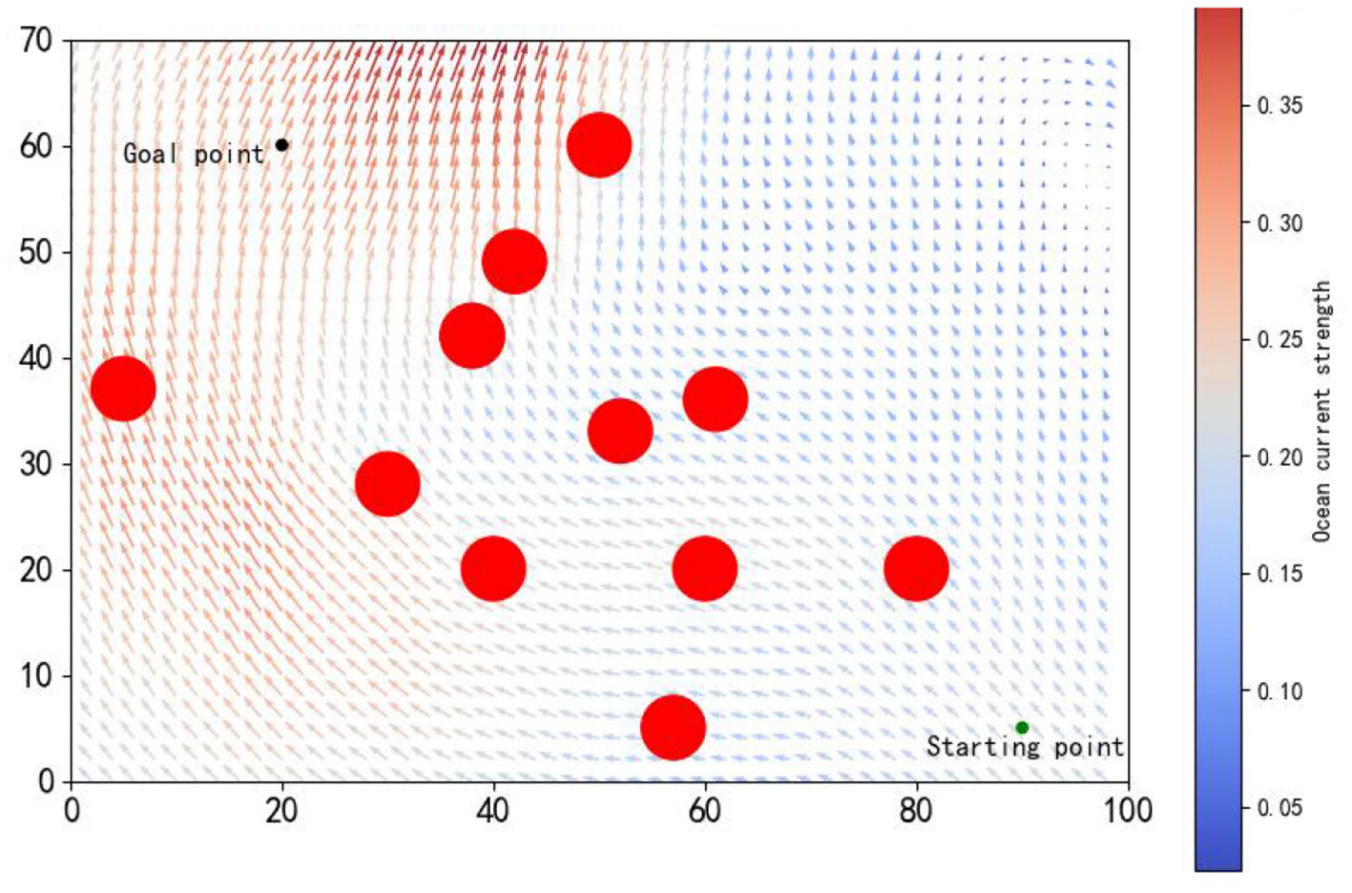
The simulated environment features a starting point (green) and an endpoint (red), with arrows indicating ocean current magnitude and direction, and longer arrows denoting stronger currents.

The training environment was established within a rectangular area of 100 nautical miles by 70 nautical miles, encompassing 11 red circular obstacles, each with a radius of 3 nautical miles, as depicted in Figure 5. Historical ocean current data from the southern region of Brazil (29.50°S to 31.83°S, 33.41°W to 37.37°W, 750 m below the sea surface) recorded on August 12, 2021 (IRI/LDEO, 2022), were chosen to simulate the ocean currents within the environment. The AUV's speed in still water was set to 1 kn, with a control interval of 0.1 h. The starting and goal points were designated as (90, 5) and (20, 60), respectively. Further training parameters are delineated in Table 1.

**Table 1:** Training parameters.

**Parameter**	**Value**
Weighting factor *k*_1_, *k*_2_, *k*_3_, *k*_4_, *k*_5_	5, –8, 3, 2, –2
Finish indicator *T*_*done*_	0 or 1
Goal reward *R*_*goal*_	50
Obstacle reward *R*_*col*_	–200
Sonar number *n*	12
Detection range *D*	3
Initial exploration ε_initial_	0.8
Final exploration ε_final_	0.01
Exploration decay constant *c*	10,000
Learning rate η	0.01
Batch size *m*	1,500
Training episode J	3,000
Replay buffer capacityN	10,000,000
Discount factor γ	0.9
Target network update frequency *f*_*t*_	5
Noisy Nets σ	0.017
Current length of replay buffer |N|	-
Current network parameters μ, σ	-
Target network parameters μ′, σ′	-

To assess the efficacy of diverse algorithms, we utilize a set of three performance indicators for the evaluation of the path planning outcomes. (1) Path length: Based on the mission, the AUV generates a series of coordinates (*P*_0_, *P*_1_, *P*_2_, …, *P*_*l*_) according to the algorithm, where *P*_0_ and *P*_*l*_ represent the start and goal points, respectively, while the points *P*_*j*_ (*j* ≠ 0, *l*) are intermediate points. Each point is represented by coordinates *P*_*j*_ = (*x*_*j*_, *y*_*j*_). The total path length *L* is calculated as follows:


(24) $$\begin{array}{c} L=\overset{l}{\underset{j=1}{\sum }}L_{euc}\left(P_{j},P_{j-1}\right). \end{array}$$

(2) Travel time: The travel time *T* represents the cumulative duration for the AUV to traverse from its origin to its destination, factoring in both the AUV's velocity and the speed of the ocean currents. The calculation is expressed as:


(25) $$\begin{array}{c} T=\overset{l}{\underset{j=1}{\sum }}\frac{L_{euc}\left(P_{j},P_{j-1}\right)}{v_{j}}, \end{array}$$

where *v*_*j*_ is the velocity of the AUV in the *j* section, combining the AUV's speed and the ocean current speed.

(3) Path smoothness rate: When the AUV travels along a smooth path, it does not need to frequently adjust its heading, reducing instability caused by heading adjustments, decreasing control complexity, and allowing the AUV to navigate more stably and efficiently. Path smoothness rate(*P*_*sm*_) is calculated as follows (Chu et al., 2023):


(26) $$\begin{array}{c} P_{sm}=\frac{\sum_{j=2}^{l-1}|\chi_{j+1}-\chi_{j}|}{l-2}, \end{array}$$

(27) $$\begin{array}{c} \chi_{j}=\arctan\left(\frac{y_{j}-y_{j-1}}{x_{j}-x_{j-1}}\right), \end{array}$$

where χ_*j*_ represents the direction of AUV travel between two path points. A smaller *P*_*sm*_ value indicates a smoother path.

### 4.2 Simulation and training process

**Figure 6:**
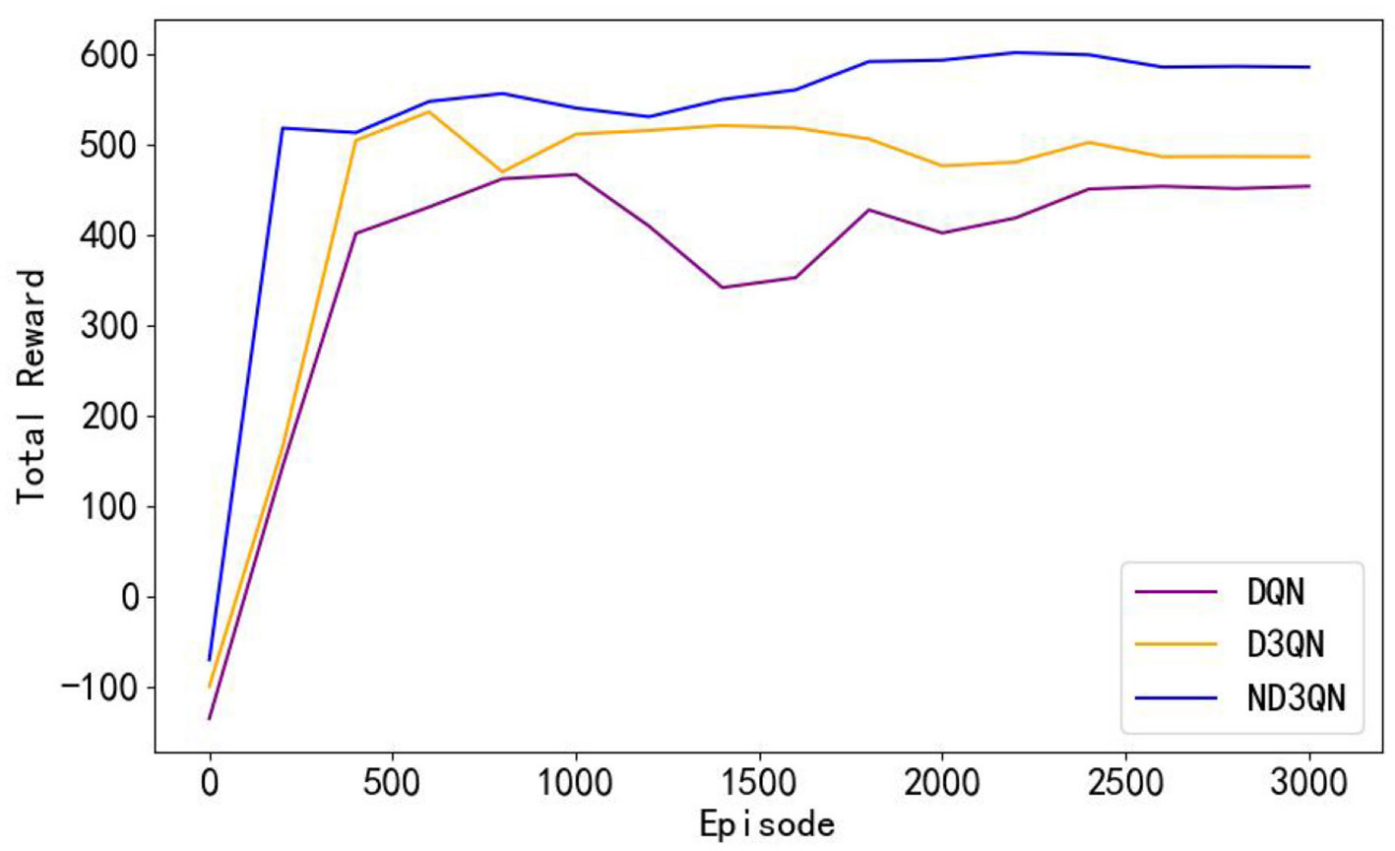
Comparison of the learning curves of different algorithms during training. Moving averages computed every 200 data points smoothed the curves.

To assess the efficacy of the ND3QN algorithm, we compared DQN (Yang et al., 2023b) and D3QN (Xi et al., 2022) in the same simulated environment. Additionally, we implemented RRT* (Chu et al., 2023) as a reference benchmark, which is an improved heuristic algorithm capable of providing immediate performance indicators for comparison with reinforcement learning algorithms without the need for training. Our proposed improved version, ND3QN, introduces a noisy network and ε-decay strategy to enhance exploration capabilities, thereby further optimizing the effectiveness of path planning. We conducted 3000 training sessions for each of these three reinforcement learning algorithms, and Figure 6 illustrates their total reward value variations during the training process, reflecting their learning progress, and performance in path planning tasks.

As shown in Figure 6, the total rewards of all algorithms steadily increase with the number of training episodes and eventually stabilize. From episodes 500 to 3,000, the performance of the algorithms remains relatively stable. Table 2 reveals that ND3QN achieves the highest average total reward (557.5526) with the lowest standard deviation (162.2245). Additionally, the difference in average total reward between ND3QN and both DQN and D3QN is statistically significant (*p***<**0.001). These findings indicate that ND3QN not only achieves higher total rewards but also outperforms the other algorithms in terms of stability. This can be attributed to the integration of a noisy network in the ND3QN algorithm, allowing the agent to dynamically adjust the noise level based on the training progress. In the early training phase, higher noise enhances exploration by increasing randomness, enabling the agent to cover a broader state space and discover high-reward strategies faster. As training progresses, the noise level gradually decreases, balancing exploration and exploitation. This helps prevent premature convergence to suboptimal solutions while maintaining stability in performance, ultimately leading to a higher and more stable total reward.

**Table 2:** Average reward, standard deviation, and statistical significance of ND3QN compared to other algorithms.

**Algorithm**	**Average reward**	**Standard deviation**	***p*-value (vs. ND3QN)**
DQN	422.8933	209.3916	**<**0.001
DDQN	502.3295	191.1955	**<**0.001
ND3QN	557.5526	162.2245	-

**Figure 7:**
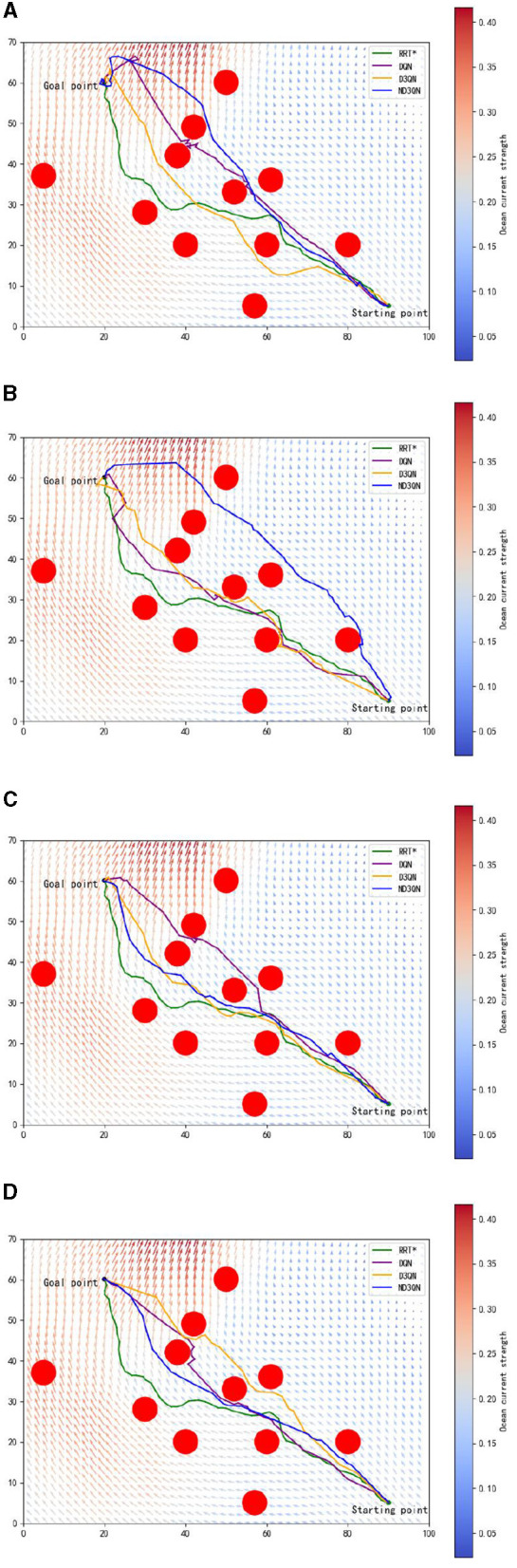
Results of different training episodes. **(A)** Results of 500 training episodes. **(B)** Results of 1,000 training episodes. **(C)** Results of 2,000 training episodes. **(D)** Results of 3,000 training episodes.

Figure 7 shows the results of DQN, D3QN, and ND3QN algorithms planning paths during training and compares the paths generated by RRT* in the same environment. As shown in Figure 7A, at the early stages of training, while DQN, D3QN, and ND3QN all successfully reach the target, the planned paths tend to be more convoluted. As training progresses, these reinforcement learning algorithms gradually learn to plan shorter and smoother paths. By the later stages of training, as depicted in Figure 7D, ND3QN not only generates paths that are shorter and smoother than those produced by the other algorithms, but it also better adapts to the direction of ocean currents, effectively leveraging them to reduce travel time.

Table 3 presents the variations in path length, travel time, and path smoothness for three algorithms during the training process. In terms of path length, ND3QN reduced the path length to 93.5870 nautical miles, which is shorter compared to DQN's 95.1229 nautical miles and D3QN's 94.1267 nautical miles. Regarding travel time, ND3QN decreased from an initial 93.2385 h to 79.4785 h, representing a reduction of approximately 9.04% compared to the RRT* algorithm (87.3821 h), which does not consider ocean currents. Compared to DQN (82.5455 h) and D3QN (82.1552 h), ND3QN reduced travel time by 3.72% and 3.26%, respectively. This improvement is mainly due to the incorporation of ocean current information into the reward function, which guides the AUV to select paths more aligned with the direction of the currents. Consequently, the AUV is able to harness the current's driving force more effectively, thereby reducing the required travel time. Furthermore, ND3QN's path smoothness improved significantly, dropping from 0.3499 to 0.1001, indicating that the paths became progressively smoother. Compared to RRT*, DQN, and D3QN, the path smoothness of ND3QN improved by 86.90%, 63.66%, and 54.45%, respectively. This improvement is due to the fact that the reward function of the ND3QN algorithm also takes into account the degree of direction change between consecutive actions, and penalizes actions with too much direction change, thereby reducing the appearance of zigzagging paths and significantly enhancing path smoothness.

**Table 3:** Performance comparison of different algorithms over training episodes.

**Training episodes**	**Algorithm**	**Path length (n mile)**	**Travel time (h)**	**Smoothness rate (rad)**
500	DQN	110.7443	100.6811	0.4737
	D3QN	109.4090	100.0773	0.3878
	ND3QN	106.6636	93.2385	0.3499
1,000	DQN	103.8093	89.4617	0.3375
	D3QN	103.7286	90.2009	0.6861
	ND3QN	102.9158	96.0647	0.3373
2,000	DQN	96.3144	84.9202	0.3678
	D3QN	97.6397	83.5286	0.3662
	ND3QN	96.0469	81.5757	0.3501
3,000	DQN	95.1229	82.5455	0.2755
	D3QN	94.1267	82.1552	0.2198
	ND3QN	93.5870	79.4785	0.1001
RRT* (Chu et al., 2023)		103.3822	87.3821	0.7641

In summary, we trained the ND3QN algorithm in a fixed obstacle environment and compared it with the RRT*, DQN, and D3QN algorithms. Through training, the agent evolved from an initial state of ignorance to ultimately mastering a more effective path-planning strategy. Compared to other algorithms, ND3QN leveraged noise networks and incorporated an ε-decay strategy to achieve adaptive exploration, thereby avoiding premature convergence to suboptimal solutions and more effectively planning superior paths.

### 4.3 Simulation in different environments

In AUV path planning, the location of obstacles is one of the key factors affecting the success rate of path planning. The AUV must detect and avoid obstacles to reach the target successfully. In the experiment presented in Section 4.2, the model gradually learned effective path-planning capabilities within the training environment. However, the model's performance in unknown environments, where obstacle positions differ from those in the training phase, remains uncertain. To assess the path-planning success rate and travel time of the ND3QN algorithm in unfamiliar and complex environments, we generated 200 test environments with varying obstacle positions. Each environment contained 30 randomly distributed circular obstacles with a radius of 3 nautical miles. To mitigate the inherent randomness caused by different random seeds, we conducted the experiments using five different random seeds. Additionally, we compared the ND3QN algorithm with the previously mentioned RRT*, DQN, and D3QN algorithms. The 200 test environments generated by these algorithms under the same random seed are identical to compare the performance of different algorithms in the same obstacle environment.

**Figure 8:**
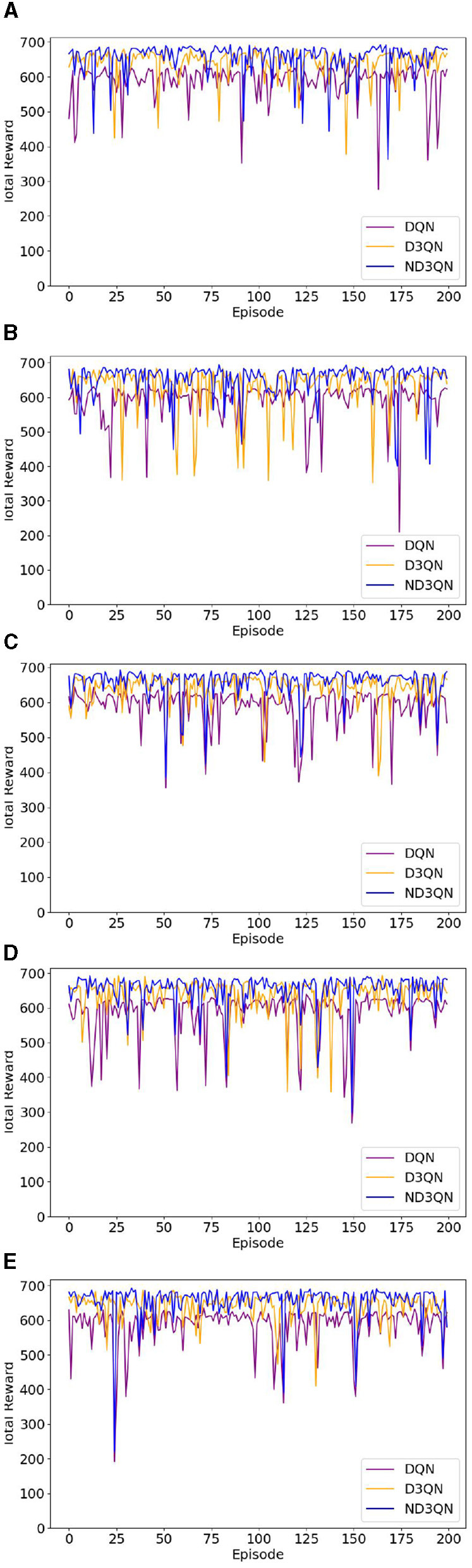
The reward values of DQN, D3QN, and ND3QN algorithms in 200 different test environments under 5 random seeds. The horizontal coordinate represents the environment number and the vertical coordinate represents the total reward value. **(A)** Results of seed = 10. **(B)** Results of seed = 30. **(C)** Results of seed = 50. **(D)** Results of seed = 70. **(E)** Results of seed = 90.

**Figure 9:**
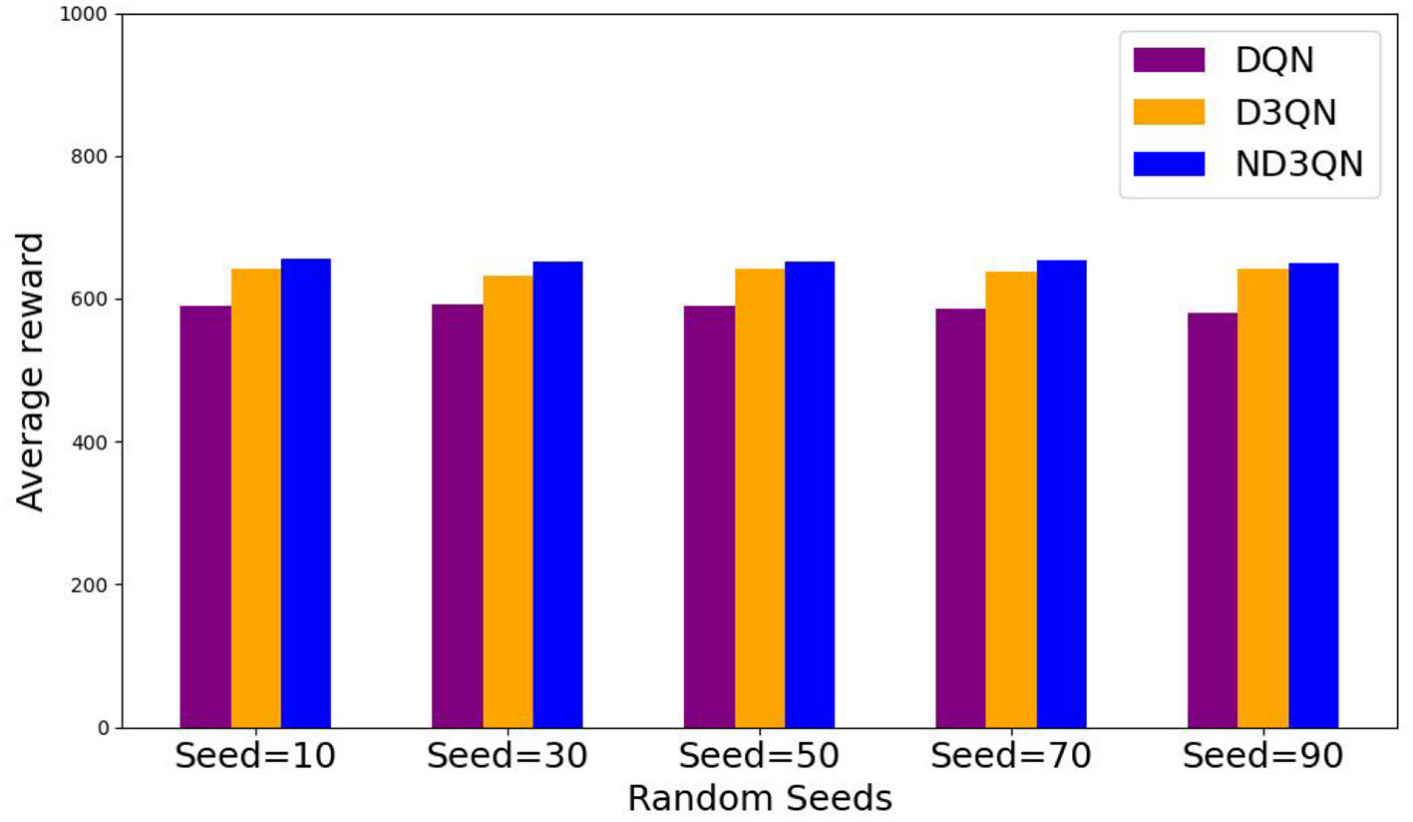
Average reward values of DQN, D3QN, and ND3QN algorithms in 200 different environments under 5 random seeds.

**Figure 10:**
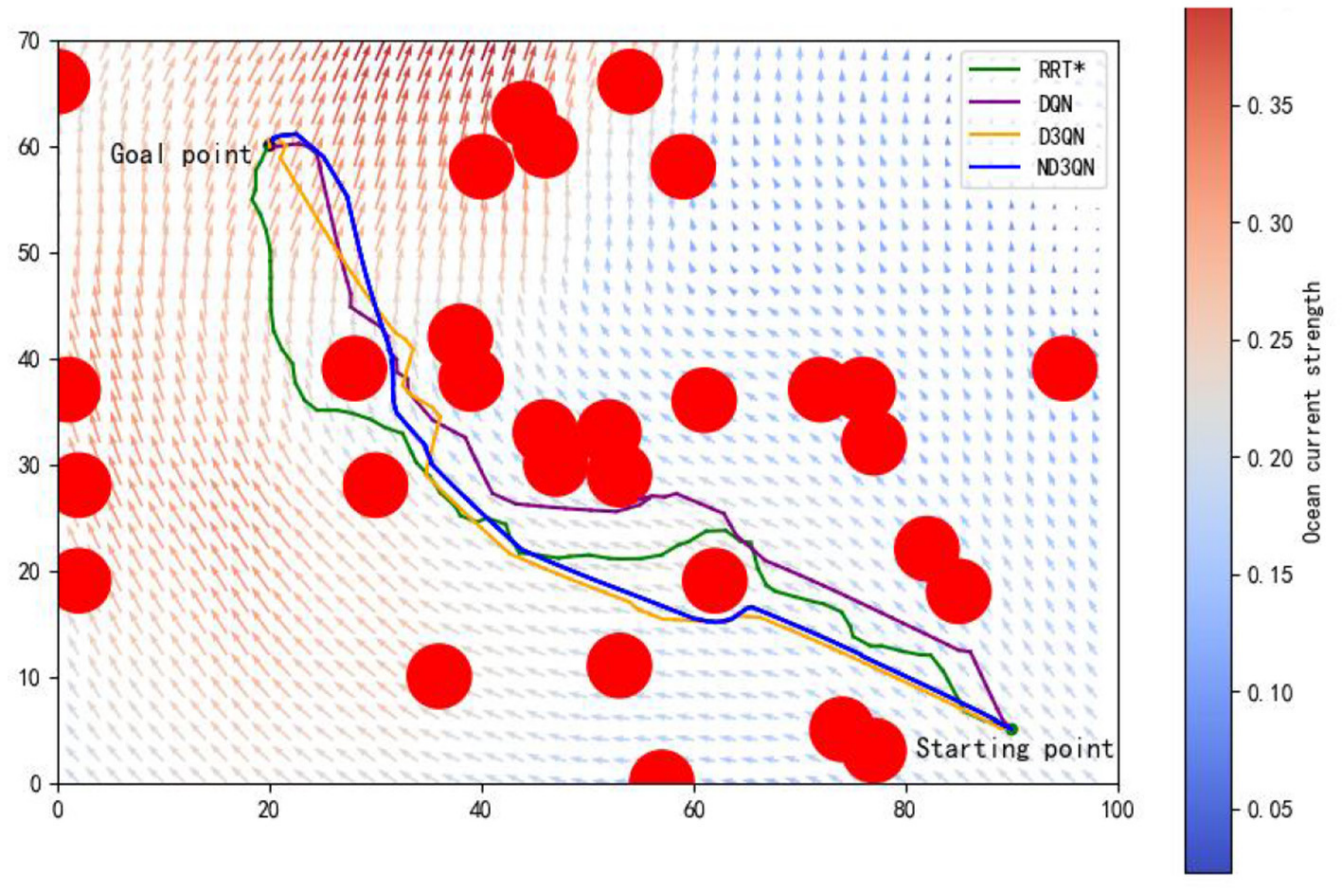
One of the path planning results from 200 different environments in one of the random seeds.

Figure 8 illustrates the total rewards obtained by DQN, D3QN, and ND3QN algorithms across 200 randomly generated environments under different random seeds. On the one hand, the total reward reflects the success rate of path planning: when the total reward in a specific environment is relatively small, it likely indicates that the AUV failed to reach its destination. Figure 8 shows that the ND3QN algorithm has fewer episodes with relatively small total reward values across 200 different environments, indicating that ND3QN successfully plans more paths and achieves a higher success rate. On the other hand, the magnitude of the total reward also represents the effectiveness of the path planning. In reinforcement learning, higher total rewards indicate that the strategy employed by the algorithm in that environment is more optimal (Sutton and Barto, 1998). Figure 9 shows the average total rewards across 200 different environments, where the results demonstrate that ND3QN consistently achieves higher average rewards compared to DQN and D3QN, further confirming ND3QN's superiority in path planning. Figure 10 presents the path planning results for one of the 200 test environments under one of the random seeds. Compared to other algorithms, ND3QN not only generates shorter and smoother paths but also aligns more effectively with the ocean's current direction, leveraging its thrust to reduce travel time.

To objectively analyze the experimental results, we conducted a quantitative assessment. Table 4 records each algorithm's success rate and, for successful paths, the total mean and standard deviation values of path length, travel time, and path smoothness. The ND3QN algorithm achieved a goal attainment rate of 93.2%, outperforming RRT*, DQN, and D3QN by 10.6%, 7%, and 4.2%, respectively. This improvement is attributed to the introduction of a noise network in ND3QN, which dynamically adjusts the exploration process, enhancing the robustness and generalization of the policy, thereby better adapting to various environments. Additionally, the obstacle avoidance rewards enable the AUV to account for potential obstacles in advance, thus improving the success rate of path planning.

**Table 4:** Comparison of path length, travel time, path smoothness and success rate of different algorithms.

**Indicators**	**Mean/Std.Dev**	RRT*	**DQN**	**D3QN**	**ND3QN**
Path length (n mile)	Seed = 10	104.4356	105.3479	99.6731	96.0253
	Seed=30	103.1272	104.2497	99.4672	96.2248
	Seed=50	103.2062	106.5713	99.5797	95.9795
	Seed=70	104.5832	106.2557	99.3446	96.3868
	Seed=90	103.8140	104.5592	99.5400	96.1649
	Total Mean	103.8332	105.3968	99.5209	96.1563
	Std.Dev	7.0096	13.0123	2.4326	2.9286
Travel time (h)	Seed = 10	90.4052	91.1377	87.5333	81.1544
	Seed=30	89.2611	90.2094	87.3685	81.3410
	Seed=50	89.5069	91.8596	87.4592	80.9236
	Seed=70	90.7199	91.2478	87.5032	81.3904
	Seed=90	89.6273	90.3334	87.4417	81.1836
	Total Mean	89.9041	90.9576	87.4612	81.1986
	Std.Dev	6.6766	13.1517	2.3595	3.1627
Smoothness rate (rad)	Seed = 10	0.8245	0.3214	0.2496	0.1036
	Seed=30	0.8139	0.3191	0.2363	0.1057
	Seed=50	0.8493	0.3940	0.2312	0.1045
	Seed=70	0.8243	0.3157	0.2429	0.1129
	Seed=90	0.8713	0.3262	0.2434	0.1155
	Total Mean	0.8367	0.3353	0.2407	0.1084
	Std.Dev	0.2261	0.1751	0.0959	0.0630
Success rate	Seed = 10	84%	86%	90%	92%
	Seed=30	84%	87%	87%	92%
	Seed=50	80%	85%	89%	94%
	Seed=70	82%	86%	89%	93%
	Seed=90	83%	87%	90%	95%
	Total Mean	82.6%	86.2%	89.0%	93.2%
	Std.Dev	0.0167	0.0084	0.0122	0.0130

For example, Seed = 10 represents the average of each metric corresponding to successful paths in 200 different environments generated using this random seed.

The analysis of variance (ANOVA) in Table 5 reveals significant differences among the algorithms in terms of path length, travel time, and path smoothness (*p***<**0.001), indicating that the path planning results differ significantly across the algorithms. The multiple comparison results in Table 6 further confirm the superiority of the ND3QN algorithm, showing significant differences in all three path quality metrics compared to RRT*, DQN, and D3QN (*p***<**0.001). For example, ND3QN's path length is 9.2405 nautical miles shorter than that of DQN (*p***<**0.001). These statistical results demonstrate the significant advantage of the ND3QN algorithm in path planning. Specifically, the ND3QN algorithm reduced path length by 7.39%, 8.77%, and 3.38% compared to RRT*, DQN, and D3QN, respectively. The average travel time of ND3QN (81.1986 h) was reduced by 9.68%, 10.73%, and 7.16% compared to RRT*, DQN, and D3QN, respectively. This indicates that even in environments with randomly generated obstacles, ND3QN can select paths more aligned with ocean currents, thereby reducing travel time. Furthermore, the average path smoothness of ND3QN improved by 87.04%, 67.66%, and 54.94% compared to RRT*, DQN, and D3QN, respectively. This improvement is attributed to the expanded action space and the incorporation of path smoothness rewards.

**Table 5:** ANOVA results of path length, travel time, and path smoothness.

**Indicators**	**Source**	**Sum of squares**	**df**	**Mean sum of squares**	**F**	**Significance**
Path length	Intergroup	47,152.39	3	15,717.46	276.13	**<**0.001
	Intragroup	199,566.04	3,506	56.92		
	Overall	246,718.42	3,509			
Travel time	Intergroup	51,912.92	3	17,304.31	303.40	**<**0.001
	Intragroup	199,962.55	3,506	57.03		
	Overall	251,875.47	3,509			
Smoothness rate	Intergroup	260.00	3	86.67	3,777.15	**<**0.001
	Intragroup	80.44	3,506	0.02		
	Overall	340.44	3,509			

**Table 6:** Results of multiple comparisons of the significance of mean differences between different algorithms.

**Indicators**	**Comparison**	**Mean difference**	**Standard error**	**Significance**
Path length	RRT^*^ vs. DQN	–1.5636^**^	0.8782	0.021
	RRT^*^ vs. D3QN	4.3123^***^	0.9357	**<**0.001
	DQN vs. D3QN	5.8759^***^	0.9028	**<**0.001
	RRT^*^ vs. ND3QN	7.6769^***^	0.8681	**<**0.001
	DQN vs. ND3QN	9.2405^***^	0.8326	**<**0.001
	D3QN vs. ND3QN	3.3646^***^	0.8930	**<**0.001
Travel time	RRT^*^ vs. DQN	–1.0535^**^	0.8802	0.025
	RRT^*^ vs. D3QN	2.4429^**^	0.9377	0.038
	DQN vs. D3QN	3.4964^***^	0.9048	**<**0.001
	RRT^*^ vs. ND3QN	8.7055^***^	0.8700	**<**0.001
	DQN vs. ND3QN	9.7590^***^	0.8344	**<**0.001
	D3QN vs. ND3QN	6.2626^***^	0.8949	**<**0.001
Path smoothness	RRT^*^ vs. DQN	0.5014^***^	0.0171	**<**0.001
	RRT^*^ vs. D3QN	0.5960^***^	0.0183	**<**0.001
	DQN vs. D3QN	0.0946^***^	0.0176	**<**0.001
	RRT^*^ vs. ND3QN	0.7283^***^	0.0169	**<**0.001
	DQN vs. ND3QN	0.2269^***^	0.0162	**<**0.001
	D3QN vs. ND3QN	0.1323^***^	0.0174	**<**0.001

The mean difference represents the mean of the former algorithm subtracted by the mean of the latter in each pairwise comparison. *, **, and *** denote significance at the 10%, 5%, and 1% levels, respectively.

In this section, the unknown environment's path planning scenario was simulated by randomly placing obstacles. The experimental results indicate that the ND3QN algorithm significantly outperforms the RRT*, DQN, and D3QN algorithms in the three evaluation metrics: path length, travel time, and path smoothness. Compared to these three algorithms, ND3QN reduced path length by approximately 3%–9%, travel time by about 7%–11%, and improved path smoothness by around 55%–88%, with an increased success rate of 4%–11%. This demonstrates the ND3QN algorithm's significant advantage in planning shorter, smoother, and more efficient paths, better able to adapt to unknown environments.

### 4.4 Comparison with other algorithms

To further validate the performance of the ND3QN algorithm, we compared it with several recently proposed path-planning algorithms. The BI-RRT* algorithm, introduced by Fan et al. (2024), improves path planning capabilities over the traditional RRT* by expanding the obstacle regions, employing a bidirectional search strategy, and utilizing cubic spline interpolation for path smoothing. Yang et al. (2023b) developed the NPDDQN algorithm, which enhances adaptability in complex environments by integrating double DQN with prioritized experience replay and multi-step reward strategies.

**Figure 11:**
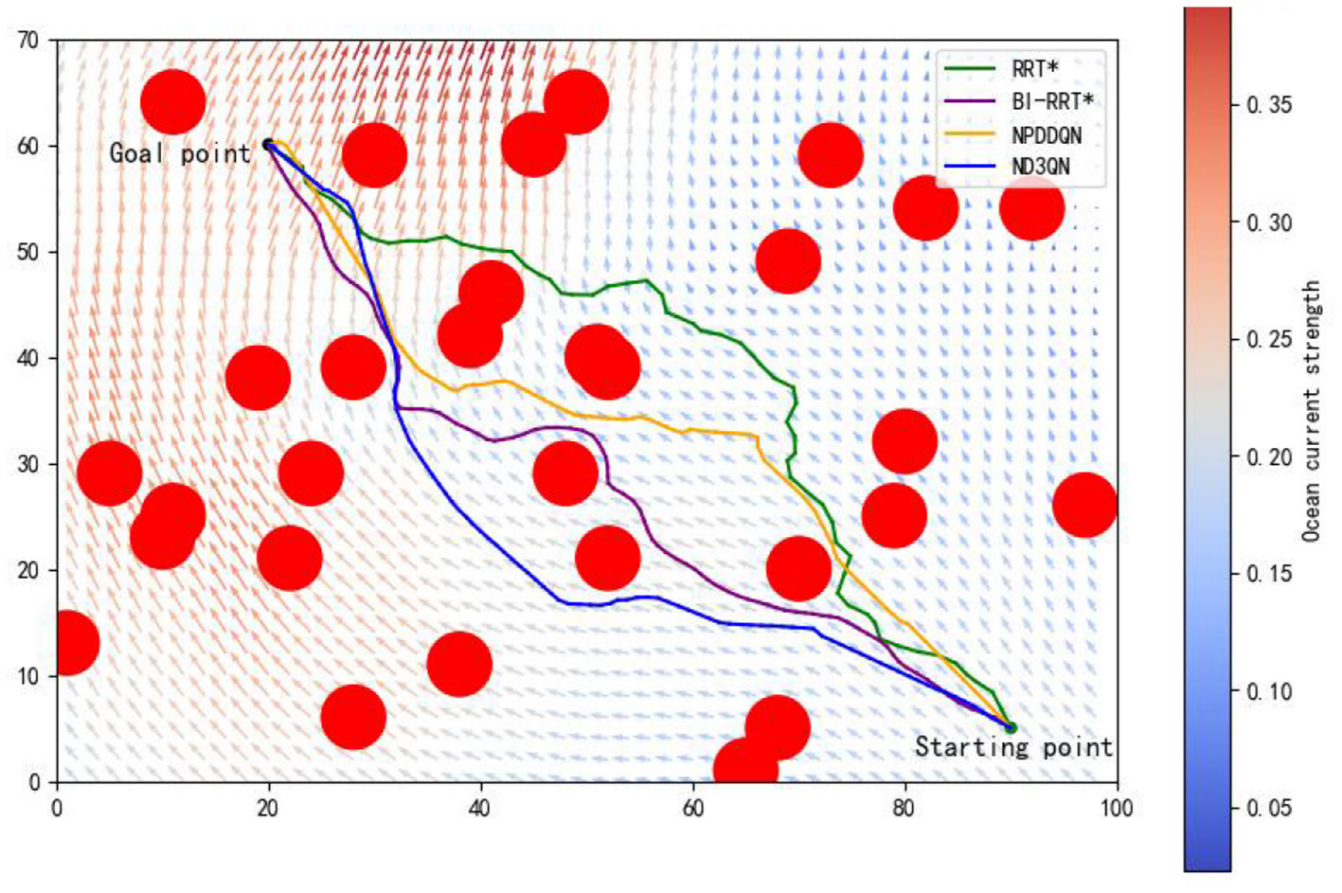
Comparison of the results of paths planned by RRT*, BI-RRT*, NPDDQN, and ND3QN algorithms.

In the same complex environment with 30 obstacles, we conducted a detailed comparison of the RRT*, BI-RRT*, NPDDQN, and ND3QN algorithms, with the experimental results shown in Figure 11 and Table 7. Specifically, ND3QN's travel time is 81.8266 h, which is 3.64 h faster than the BI-RRT* and 2.2311 h faster than the NPDDQN. Regarding path smoothness, BI-RRT* improves path smoothness considerably through interpolation techniques, outperforming RRT*. However, ND3QN achieves the lowest smoothness value, indicating that it generates the smoothest paths among all compared algorithms. These results demonstrate ND3QN's superior performance in terms of path length, travel time, and path smoothness, further validating its potential for underwater path planning tasks.

**Table 7:** Performance comparison of RRT*, BI-RRT*, NPDDQN, and ND3QN algorithms.

**Algorithm**	**Path length (n mile)**	**Travel time (h)**	**Smoothness rate (rad)**
RRT*	104.1384	90.6913	0.9181
BI-RRT* (Fan et al., 2024)	99.7849	85.5106	0.2650
NPDDQN (Yang et al., 2023b)	98.2697	84.0577	0.3983
ND3QN	98.0723	81.8266	0.1578

### 4.5 Results in real terrain environment

**Figure 12:**
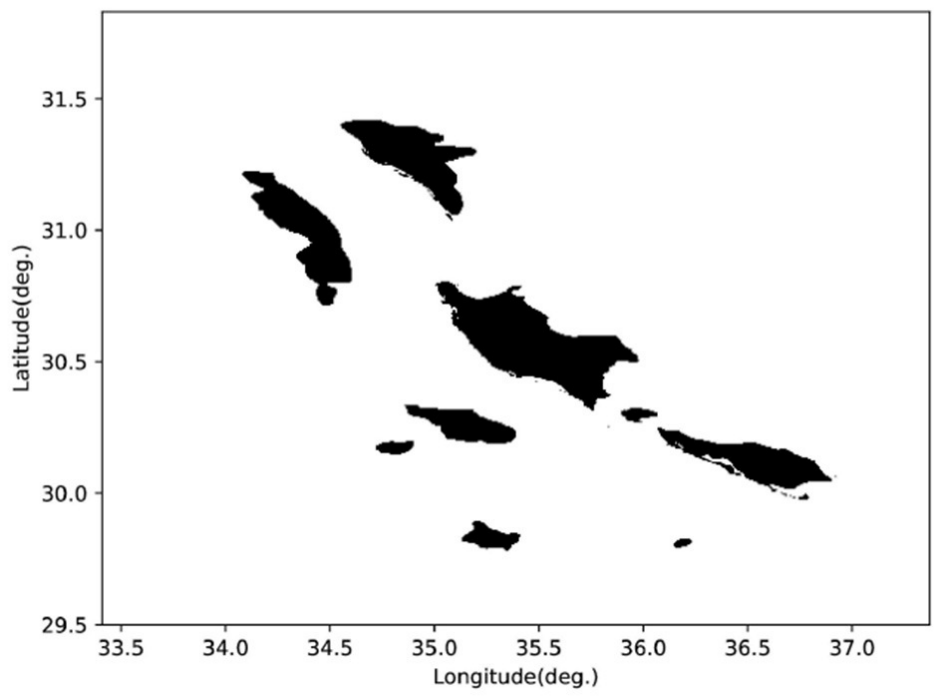
Real terrain area: 29.50°S to 31.83°S, 33.41°W to 37.37°W, 750 m below the sea surface.

**Figure 13:**
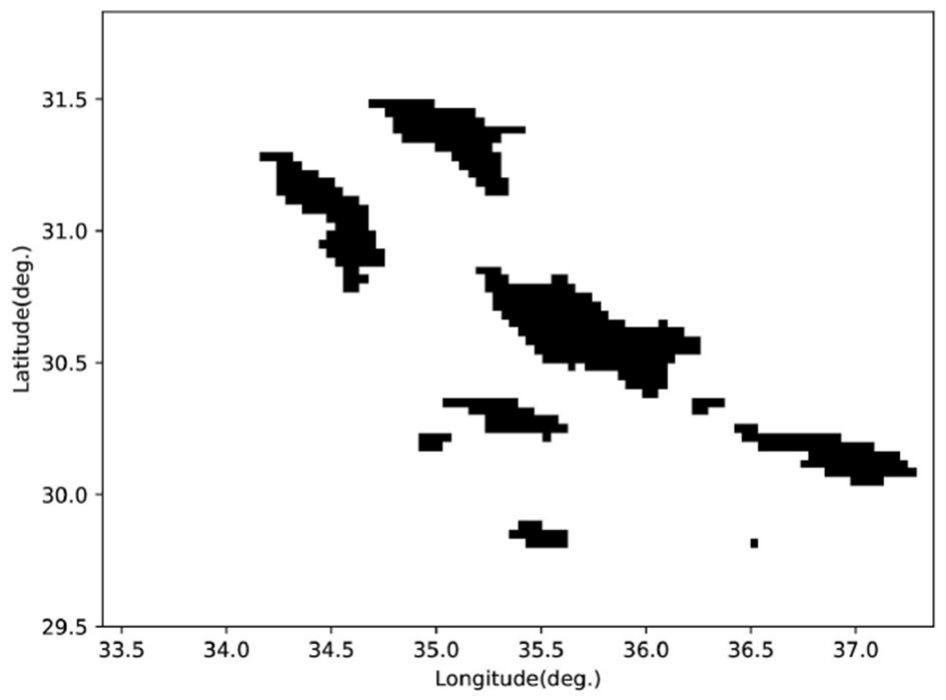
Grid maps compressed from real terrain.

**Figure 14:**
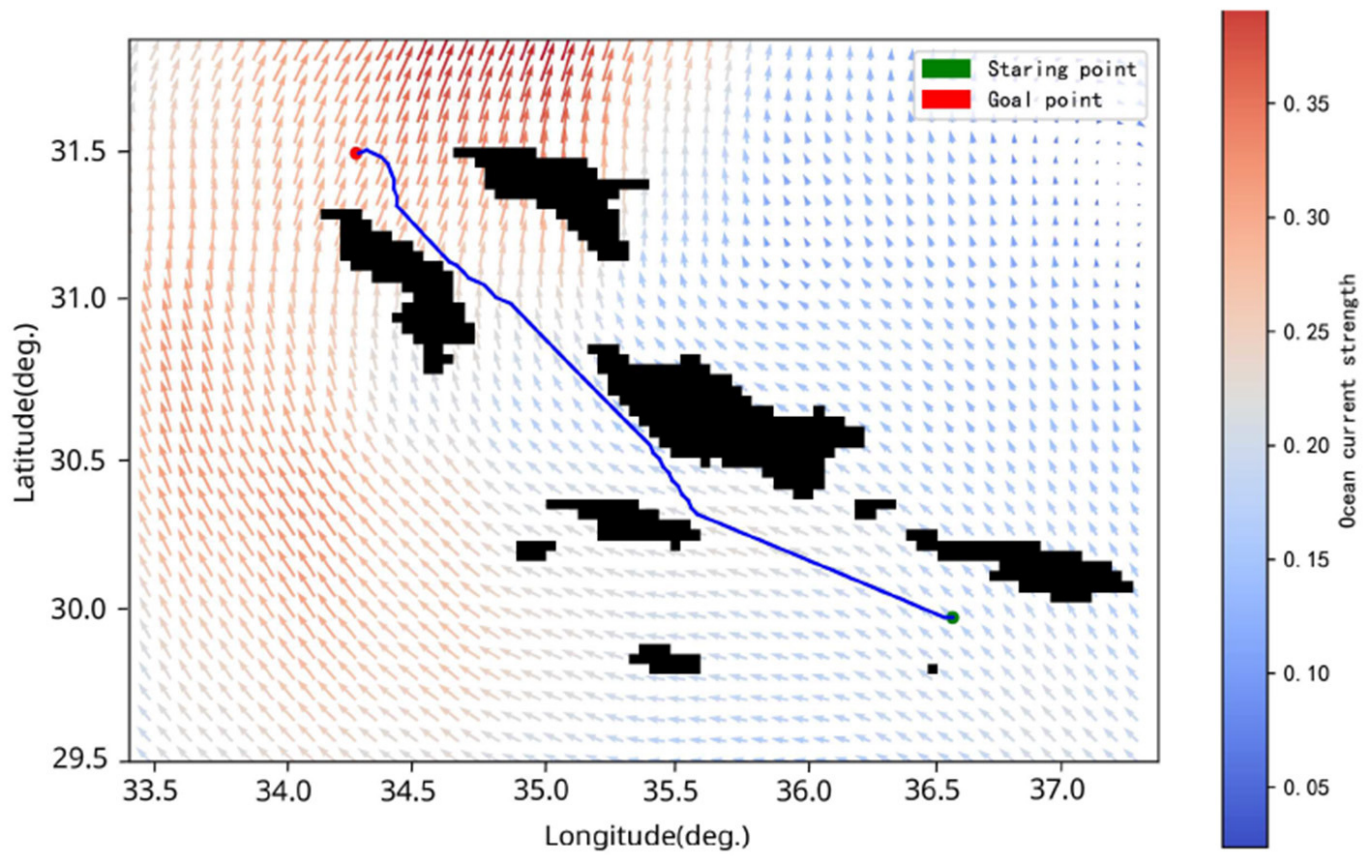
Planned paths from (30.04°S, 36.55°W) to (31.49°S, 34.26°W). The green symbol indicates the starting point, and the red symbol signifies the endpoint.

We selected a real terrain area (GEBCO Compilation Group, 2020), as shown in Figure 12. To facilitate the calculation of the detection distance matrix through the simulated sonar model, we compressed the real terrain into a grid map, as illustrated in Figure 13. We configured the AUV to cruise at 4 kn, with the starting point at (30.04°S, 36.55°W) and the goal point at (31.49°S, 34.26°W). Despite the increased difficulty due to the larger search space, our algorithm was still able to plan a time-efficient path. Figure 14 shows the planned path, with a length of 276.67 nautical miles and a time cost of 34.03 h. In the real environment, the terrain is more complex compared to the circular obstacles used in the simulation. However, by inputting the detection distances obtained from the simulated sonar into the agent as state information, the AUV arrives at the objective site without incident. This demonstrates that our algorithm can handle missions in real ocean environments, showcasing its feasibility in practical scenarios.

## 5 Conclusion

In this work, we present a novel path-planning method, ND3QN, to solve the problem of existing reinforcement learning algorithms' inadequate exploration-exploitation balance during AUV path-planning. This algorithm extends the action space of AUV, providing up to 16 finely tuned action modes, enabling AUV to adapt more flexibly to dynamic changes in complex ocean currents. Then, we design a composite reward function that comprehensively considers factors such as distance, obstacles, ocean currents, path smoothness, and the number of steps to guide AUV in finding paths with shorter travel times and smoother paths. Additionally, the ND3QN algorithm introduces noisy networks based on traditional D3QN, combined with the ε-decay strategy, effectively balancing the exploration and exploitation trade-off in AUV path planning. Comparing the ND3QN to the DQN and D3QN algorithms, experimental results show that the ND3QN approach displays faster convergence and greater convergence values during training. In order to test the adaptability of the algorithm in different environments, we randomly generated 200 environments with different obstacle positions to conduct simulation experiments. At the same time, different random seeds were changed to mitigate the inherent randomness. The experimental results show that the success rate of ND3QN reaches 93%, which is 4%–11% higher than that of the RRT*, DQN, and D3QN algorithms. Furthermore, the average travel time of ND3QN is reduced by approximately 7%–11%, while the path smoothness is improved by about 55%–88%. The path-planning capability of the ND3QN algorithm is also validated in real terrain. These results provide more evidence of the ND3QN algorithm's excellence in enhancing adaptability to different unknown environments.

The ND3QN algorithm aids in efficiently and safely planning paths for the AUV, enabling it to avoid obstacles and utilize ocean currents effectively. This algorithm can be used in environmental monitoring, deep-sea exploration, and seafloor mapping. However, deep reinforcement learning-based methods typically require substantial data and computational resources during training. Therefore, it is necessary to optimize the training methods and model architecture further. Additionally, data collected by underwater sensors may contain noise, making it challenging to obtain accurate environmental information (such as currents, terrain, and obstacles), which could impact the algorithm's performance.

In future research, we will further consider continuous angular output as navigation direction and improve the ND3QN algorithm to make it suitable for path planning in 3D marine environments. In addition, we will study the cooperative path planning problem for multiple autonomous underwater vehicles (AUVs).

## Data Availability

The original contributions presented in the study are included in the article/supplementary material, further inquiries can be directed to the corresponding author.
